# Energy transfer within the hydrogen bonding network of water following resonant terahertz excitation

**DOI:** 10.1126/sciadv.aay7074

**Published:** 2020-04-24

**Authors:** Hossam Elgabarty, Tobias Kampfrath, Douwe Jan Bonthuis, Vasileios Balos, Naveen Kumar Kaliannan, Philip Loche, Roland R. Netz, Martin Wolf, Thomas D. Kühne, Mohsen Sajadi

**Affiliations:** 1 Department of Chemistry, University of Paderborn, Paderborn, Germany.; 2 Fritz-Haber-Institut der Max-Planck-Gesellschaft, Berlin, Germany.; 3 Department of Physics, Freie Universität Berlin, Berlin, Germany.; 4 Institute of Theoretical and Computational Physics, Graz University of Technology, 8010 Graz, Austria.

## Abstract

Energy dissipation in water is very fast and more efficient than in many other liquids. This behavior is commonly attributed to the intermolecular interactions associated with hydrogen bonding. Here, we investigate the dynamic energy flow in the hydrogen bond network of liquid water by a pump-probe experiment. We resonantly excite intermolecular degrees of freedom with ultrashort single-cycle terahertz pulses and monitor its Raman response. By using ultrathin sample cell windows, a background-free bipolar signal whose tail relaxes monoexponentially is obtained. The relaxation is attributed to the molecular translational motions, using complementary experiments, force field, and ab initio molecular dynamics simulations. They reveal an initial coupling of the terahertz electric field to the molecular rotational degrees of freedom whose energy is rapidly transferred, within the excitation pulse duration, to the restricted translational motion of neighboring molecules. This rapid energy transfer may be rationalized by the strong anharmonicity of the intermolecular interactions.

## Teaser

Energy flow in the hydrogen bonding network of water is traced by resonant terahertz excitation and off-resonant optical probing.

## INTRODUCTION

Water is a major substance on the earth surface. Its diverse anomalous properties make life on our planet viable. Notably, its large heat capacity turns oceans and seas into giant heat reservoirs for regulating the earth climate. In living organisms, the same property makes water a superb thermal buffer for the function of biochemical reactions (*1*, *2*). These thermodynamic peculiarities are commonly attributed to water’s ability to form an intermolecular complex network, which is based on thermally fluctuating hydrogen (H) bonds. As each water molecule forms on average close to four H-bonds with ~1-ps lifetime in an almost tetrahedral configuration (*3*), the three-dimensional network of H-bonded water molecules encompasses complex collective and/or cooperative intermolecular degrees of freedom with a very diverse dynamics (*4*, *5*).

The molecular dynamics (MD) associated with this network, including the restricted translations and rotations as well as the diffusive motions, cover an exceptionally broad frequency range, with a bandwidth of more than 1000 cm^−1^. These spectrally broad intermolecular degrees of freedom may then serve as a heat sink with abundant pathways for the accommodation/dissipation of deposited excess energy in water (*6*), explaining its large heat capacity (*7*). The extension of the intermolecular modes to high frequencies makes it also an ideal/efficient thermal bath for ultrafast relaxation of energy from vibronically hot (bio-) molecules, thereby avoiding their permanent thermal damage (*8*). To elucidate the molecular mechanism of the energy dissipation in water and understand the role of collective intermolecular motions in this process, the time scale of energy dissipation and also the strength of intermolecular interactions should be determined by experiments.

While linear-type spectroscopic methods, such as dielectric relaxation, determine the polarization decay of the infrared (IR)–active modes of liquids, nonlinear IR spectroscopy has extensively been used to provide complementary microscopic insights into the accompanying energy dissipation processes. For example, the O─H stretch vibration has been used as a local probe to interrogate the dynamics of its surrounding (*9*, *10*). Previous studies using this approach proposed the dipole-dipole interaction to be the main mechanism of the vibrational energy transfer in water (*9*) and determined the time scale of this process to be <100 fs (*10*). Moreover, ultrafast (sub–100 fs) energy transfer from the OH bending vibration to the librational (hindered rotational) motion has also been resolved (*11*).

However, despite these efforts, there are still various open questions regarding the energy flow in the H-bond network. For example, to what extent do intermolecular modes and processes contribute to the energy transfer within the H-bond network of water? What is the time scale for the energy transfer between these motions, and how strongly they are coupled? We believe that a more accurate understanding of the energy dissipation process in water will emerge by direct interrogation of the low-frequency intermolecular degrees of freedom. Because the spectral fingerprint of the intermolecular H-bonding dynamics lies in the terahertz (THz) frequency range, it is promising to resonantly pump the low-frequency collective modes/processes of water with a THz pulse and watch the response of the system in real time.

This method has already provided insights into intramolecular mode coupling in halogenated liquids (*12*) and into the resonant coupling of THz radiation to permanent molecular dipoles in various polar liquids (*13*) and has indicated that the response of water cannot be described by Langevin-type molecular rotational dynamics (*14*). MD simulations also determined the strength of the required electric field for molecular alignment in water (*5*) and showed the strong frequency dependence of its THz-induced temperature jump (*15*, *16*). In principle, the main merit of this method is that the original energy recipient mode or process can be readily assigned, and thus, the time scale and the pathways of energy flow into intermolecular degrees of freedom can be analyzed. These studies may eventually map out the complex energy potential surface of the H-bonded network of water and enable us to model the structural dynamics of water (*17*).

Here, we resonantly excite the collective rotational degrees of freedom of water with intense THz pulses and probe the resulting optical anisotropy in a THz Kerr effect (TKE) configuration (*18*). To reveal the origin of the resulting response, we perform complementary experiments, including the temperature-dependent TKE of liquid water, TKE of water vapor, and the optical Kerr effect (OKE) of liquid water. We also perform ab initio MD (AIMD) and force field–based MD (FFMD) simulations under the effect of the same THz field to gain deeper insights into the process of intermolecular energy transfer in water. In the AIMD simulations, the interatomic interactions are computed “on-the-fly” by electronic structure calculations.

## RESULTS

### Experimental

**Fig. 1:**
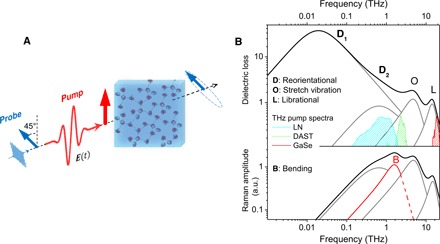
Dynamic TKE. (**A**) An intense THz pump pulse induces optical birefringence in water. The effect is monitored by an optical probe pulse that becomes elliptically polarized upon traversing through the medium. (**B**) Equilibrium dielectric loss (Im ε) and incoherent Raman spectra of water (*26*). Two Debye processes and two vibrations (network stretch vibrations and single-molecule hindered rotation, libration) are typically sufficient to fit the dielectric spectrum of water (*25*). The Raman spectrum of water lacks the first Debye process (*26*), but the H-bond bending vibration (red line) gains a significant amplitude. The spectra of the excitation THz fields at ~1, ~3, and 19 THz are indicated by the cyan, green, and red dashed areas, respectively. a.u., arbitrary units.

A schematic of the TKE experiment is shown in Fig. 1A. An intense linearly polarized THz electric field excites double-distilled water (*19*). The pump-induced optical birefringence ∆*n*(*t*) is then measured by a probe pulse (800 nm, 2 nJ, 8 fs) whose linear polarization acquires ellipticity by traversing the sample.

In a first set of experiments, water is excited in a top-open bucket to directly compare its TKE signals in the vapor and the liquid states. In this experiment, the sample is excited with the THz pump pulse centered at 0.7 THz, and its temperature is raised from 283 to 340 K. The density of the vapor on top of the bucket is changed with temperature.

To precisely obtain the TKE response of liquid water with no vapor contribution, a liquid water film (thickness of 100 μm) is held between a rear glass window and a 150-nm-thick silicon nitride (SiN) membrane as the entrance window (*20*). These thin windows exhibit a negligibly small Kerr signal (*10*) and ease challenges for separating the liquid response from that of the windows (*14*). Temperature-dependent TKE signals (interval of 274 to 345 K) are also obtained for liquid water in the cell.

We also compare the THz field–induced optical birefringence of liquid water and that induced by an optical pump pulse. Both optical and THz excitations are conducted in the same setup under otherwise identical conditions.

In the TKE process of polar liquids, the THz pulse resonantly drives the IR-active intermolecular degrees of freedom, and the optical probe pulse interrogates the dynamics of the Raman-active modes/processes. To facilitate the interpretation of our observed signals, the dielectric loss (Im ε) and the incoherent Raman spectrum of liquid water are provided in Fig. 1B (*13*). The amplitude spectra of the three THz pulses used in this study are also shown by dashed areas.

The lowest-frequency THz pulse at ~0.7 THz (cyan area) is generated by optical rectification of laser pulses (center wavelength, 800 nm; pulse duration, 350 fs; pulse energy, 4 mJ; repetition rate, 1 kHz) from an amplified laser system in a 1.3 mole percent MgO-doped stoichiometric LiNbO_3_ crystal (LN) with the tilted pulse front technique (*21*). With the single-cycle waveform, its THz electric field has a strength of ~2 million volts (MV)/cm. The intense THz pulse at about 3 THz (green area) with a field strength of ~1 MV/cm is generated by optical rectification of IR pulses (1300 nm, 60 fs, 1 mJ) in the organic crystal 4-*N*,*N*-dimethylamino-4′-*N*′-methyl-stilbazolium tosylate (DAST) (*22*). The third THz pulse at ~19 THz with ~1-μJ energy is generated by difference frequency mixing of two IR pulses (~1-mJ pulse energy, center wavelengths of 1280 and 1400 nm, 50 fs) in a nonlinear-optical GaSe crystal (*23*).

### TKE of water

**Fig. 2:**
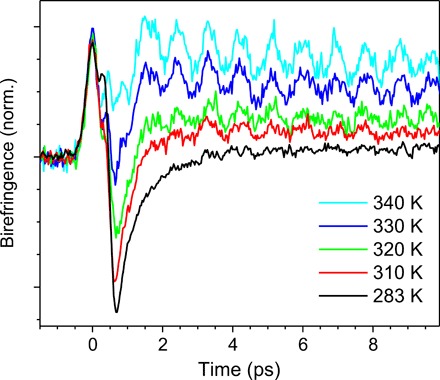
Dynamic Kerr effect of water. In a vertical configuration, the THz pump and optical probe pulses propagate into a top-open bucket of water. The ratio of vapor to liquid in the path of the two beams varies by temperature. The vapor (liquid) signal is dominant at higher (lower) temperatures. The strong oscillations indicate single-molecule coherent rotational motion of water vapor. A remarkable effect, pertinent to the current study, is the bipolar TKE signal of liquid water.

We start with the TKE signal in the top-open bucket of water after its excitation with the pulse at ~0.7 THz. As shown in Fig. 2, at high temperatures where the mixture contains large amount of water vapor, we resolve a unipolar TKE signal whose tail is modulated by a strong oscillation, a clear signature of the rotational transition of free water molecules. At lower temperatures, a remarkable effect is revealed; the TKE signal flips sign and becomes bipolar.

**Fig. 3:**
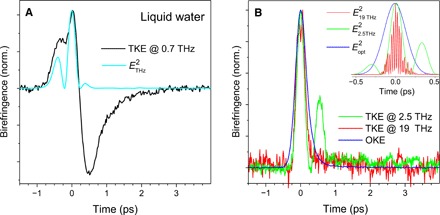
Impact of THz pump frequency on TKE signal of liquid water. (**A**) Bipolar TKE signal of water after pumping at 0.7 THz. The square of the THz field is given by the cyan line. (**B**) The TKE signals of water pumped at ~3 and ~19 THz and its OKE signal all have unipolar shape. All the signals are obtained at room temperature, where a 100-μm-thick liquid water film is held between a rear glass window and a 150-nm-thick SiN membrane as the entrance window.

The measured TKE signal of water (pumped at ~1 THz) in the cell and at room temperature is shown in Fig. 3A, a bipolar signal whose tail relaxes with 500 ± 20 fs. Our complementary experiments declare that the latter signal remains intact when the THz field is reversed, in line with the quadratic dependence of the TKE signal on the THz field amplitude (see fig. S1). The transient birefringence of water following optical excitation as well as the excitations at ~19 and ~3 THz are shown in Fig. 3B. All signals are unipolar with relatively weak relaxation tail. The comparison between the dynamic Kerr signals in Fig. 3 reveals three distinct features of the TKE signal of liquid water pumped at ~1 THz:

1)Bipolarity: The TKE response of liquid water excited at about 1 THz is bipolar, in stark contrast to the TKE signal of water vapor, the OKE signal, and the TKE signals of water pumped at ~3 and ~19 THz. The bipolar TKE signals of liquids have so far been observed only in water and n-alcohols (*13*, *24*).

2)Relaxation: The tail of the TKE signal relaxes with a time constant of ~0.5 ps. To determine this time constant, we phenomenologically modeled the TKE signal of water by convoluting two exponential functions with the assumed instantaneous electronic response of water, estimated by the TKE signal of a thin diamond plate. As illustrated in fig. S2, two exponential components with opposite signs and decay times of ~0.12 (green line) and ~0.5 ps (red line) can fit the experimental result reasonably well (magenta line). The discrepancy at the leading edge of the THz pulse most likely arises from the dispersion of water, which was neglected in the modeling. As the faster 0.12-ps component overlaps with the instantaneous electronic response, we focus in the current study on the 0.5-ps component. Note also that, as we use an ultrathin cell window (150-nm-thick SiN membrane), the measured TKE signal can be uniquely assigned to the liquid response. For thick windows, subtraction of the window response from the liquid response is essential and often a technical challenge, which may easily lead to the extraction of different relaxation time constants from the measured signal (*10*).

3)Enhancement: Relative to the amplitude of the feature around time zero, which resembles the THz electric field square, the bipolar TKE signal of water has an enhanced amplitude. In both the off-resonant optical excitation and the resonant excitations at ~19 and ~3 THz, the nuclear part of the dynamic Kerr signals has relatively small amplitudes.

## DISCUSSION

### Nature of the excited modes and processes

As shown in Fig. 1B, the dielectric and Raman spectra of liquid water are typically presented by two vibrations and two relaxation processes (*25*). At high frequencies above 10 THz, single-molecule hindered rotation (libration) is the main contribution to the inter-MD of water (*25*, *26*). The THz pump at ~19 THz resonantly excites this mode.

At lower frequencies at about 6 THz, there is a relatively strong contribution of the H-bond stretch vibration. With a resonance frequency of Ω_O − O_/2π ≈ 200 cm^−1^ and a damping rate of γ_O − O_ ≈ 180 cm^−1^, the stretch vibration is believed to be the result of the charge delocalization along the H-bonds (*27*). Further down at about 2 THz, the lateral motion of adjacent water molecules perpendicular to the H-bonds, namely, the H-bond bending, has a pronounced contribution in the Raman spectrum of water, while it has a negligibly small amplitude in the dielectric response of water (*27*, *28*). This band can be modeled by a Lorentzian with a resonance frequency of Ω_B_/2π ≈ 50 cm^−1^ and a damping rate of γ_B_ ≈ 115 cm^−1^ (*26*). The THz pump at about 3 THz is expected to more effectively excite these two translational modes.

The very low frequency region of water dynamics is typically fit by two Debye processes. The slowest Debye process D_1_ with the relaxation time τ_D_1__ ≈ 9 ps has a pronounced presence in the dielectric spectrum of water, while its Raman contribution is negligible (*26*). Additional Debye D_2_ is also needed to properly fit the dielectric susceptibility of water; however, the reported relaxation time of this process is very diverse ranging from τ_D_2__ ~1.2 ps (*29*) to ~0.25 ps (*25*). Note that although the microscopic origin of the relaxation processes of water is under debate, there is a general consensus of their association with rotational degrees of freedom.

### Optical birefringence

Because of the action of the pump field, polarized along *x* (see Fig. 1A), the probe pulse, polarized at 45° relative to *x*, encounters a transient difference Δ*n* = *n_x_* − *n_y_* between the refractive indices along *x* and *y* directions. The resulting birefringence is given by (*30*)

$$\Delta n\propto \langle \Delta \Pi_{\mathrm{xx}}-\Delta \Pi_{\mathrm{yy}}\rangle$$ (1)

where 〈. 〉 denotes ensemble average and ΔΠ*_ij_* is the pump-induced change in the collective electronic polarizability tensor element Π*_ij_*. Here, Π refers to the liquid phase and contains contributions from interactions/collisions between the molecules in the condensed phase. The variation ΔΠ can, in principle, be written as a sum ΔΠ^M^ + ΔΠ^I^ whose two contributions arise, respectively, from intrinsic molecular polarizability Π^M^ and the intermolecular interactions and collisions Π^I^ (*31*).

ΔΠ^M^ characterizes the degree of anisotropy of the unperturbed Π and is usually labeled Δα for single gas-phase molecules (*30*). Averaging ΔΠ^M^ over all molecules according to Eq. 1 yields an expression for Δ*n*_rot_ that scales with the degree of molecular alignment 〈P_2_(cosθ)〉 = 〈3cos^2^θ − 1〉/2 and the molecular polarizability anisotropy ΔΠ^M^, i.e., Δ*n*_rot_ ∝ ΔΠ^M^ 〈P_2_(cosθ)〉. The averaged ΔΠ^I^ makes another contribution to the transient birefringence and arises from directly or indirectly pump-induced changes in the collision-induced polarizability. In the following, we discuss which of the two contributions can explain the bipolar TKE signal of water.

### Single-molecule TKE response

Although the purpose of this work is to understand the collective dynamics of liquid water, it is useful to discuss the TKE signal of single water molecules in the gas phase. In the TKE process of polar molecules, the molecular alignment is achieved by the coupling of the THz electric field and the molecular permanent dipoles (*32*). Therefore, the induced optical birefringence Δ*n*_rot_ is given by the product of Δα and the alignment factor 〈*P*_2_( cos θ(*t*))〉; thus, Δ*n*_rot_ scales linearly with ∆α (*33*). Accordingly, the unipolar and positive signal of water vapor shown in Fig. 2 implies that ∆α_gas_ > 0. Note also that ∆α in the TKE process equals the difference between the polarizability component along the molecular dipole axis (*z* axis) and the polarizability average of the components in the plane perpendicular to the dipole axis, i.e., $∆\alpha =\alpha_{\mathrm{zz}}-\frac{\left(\alpha_{\mathrm{xx}}+\alpha_{\mathrm{yy}}\right)}{2}$ (*33*).

Although the gas-like rotation of single water molecules is fully damped in the liquid, it exists as the hindered rotation at frequencies above ~10 THz. Thereby, the unipolar TKE signal of water from the THz pump at ~19 THz (Fig. 3B) directly addresses the positive sign of the polarizability anisotropy of water in the liquid phase. This finding is also corroborated by ab initio calculations. Bosma *et al*. (*34*) showed that the Raman spectrum of liquid water can be reproduced with a polarizable model of water. In their model, the polarizability of molecules is more anisotropic than that in the gas phase. In a more recent ab initio theoretical study, Ge and Lu (*35*) considered the effect of the charge transfer and the local field of the H-bond network and drew the same conclusion. In both studies, the polarizability of water along the dipole axis is larger than the average of the two other components such that ∆α_liquid_ > 0.

The latter discussion declares that the single-molecule rotational dynamics cannot explain the bipolar shape the TKE signal of liquid water, in contrast to the conclusion drawn in (*14*). In the following, we scrutinize the THz pump effect with MD simulations.

### THz-induced molecular orientation and alignment

**Fig. 4:**
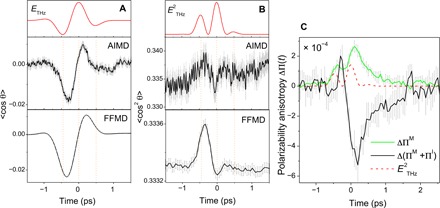
Simulated molecular orientation and alignment and polarizability anisotropy relaxation. The orientational dynamics of water molecules after THz excitation obtained from both AIMD and FFMD simulations. The θ in *y* axis is the angle between the water bisector and the THz electric field polarization axis for (**A**) the molecular orientation and (**B**) the molecular alignment. The angle brackets denote the ensemble averages. Top panels show the THz electric field and the THz intensity. Note that FFMD simulations give rise to the results with much higher signal-to-noise ratio, because 5000 trajectories are averaged for 5360 water molecule per trajectory. The AIMD and FFMD difference in the amplitude of 〈cos^2^θ〉 may be due to their differences in the microscopic water dynamics. However, with only 128 molecules in the simulation box of the AIMD simulations, the corresponding signal in (B) suffers from a large noise, as the equilibrium fluctuations of 〈cos^2^θ〉 are much larger than the signal. We also attribute the feature, resembling a slow rise in AIMD result of 〈cos^2^θ〉 to the same noise issue. (**C**) Temporal evolution of the total polarizability anisotropy of water ΔΠ^M^ + ΔΠ^I^ (in ${\overset{{}^{\circ}}{A}}^{3}$), computed from FFMD using the DID model, is compared with that of single-molecule intrinsic polarizability ΔΠ^M^ (green line).

We first calculate the degree of molecular orientation as an ensemble average of the angle between the THz electric field (at ~1 THz) and the molecular bisector. The results of our AIMD and FFMD simulations are given in Fig. 4A. They both show a discernible orientation 〈cosθ(*t*)〉 of water molecules whose patterns follow the THz waveform with a small phase shift. Here, we would like to emphasize that both MD simulations show that the deviation of 〈cosθ(*t*)〉 from its equilibrium value is small and about 2%. In addition, it is important to mention that this is not due to a few undercoordinated water molecules that are reorienting with the field, but rather, all the water molecules regardless of the number of their H-bond partners are participating in the reorientation process.

Likewise, we simulate the degree of molecular alignment 〈cos^2^θ(*t*)〉. As shown in Fig. 4B, the temporal pattern of 〈cos^2^θ(*t*)〉 manifests an ultrafast dynamics of a rise and a decay, almost fully within the temporal duration of the THz intensity profile (see also fig. S3). Note that the calculation of the orientation and the alignment of single water molecule tend to merely show the signature of the response at the single-molecule level; hence, it does not imply that the dynamics of water at this spectral region is causally governed by the single-molecule rotational motions. The fact that water has a large Kirkwood factor already indicates the collective nature of water’s reorientation dynamics at the low-frequency region (*36*).

### Simulated THz-induced optical birefringence

To shed some light on the origin of the bipolar TKE response of water, we directly calculate the THz electric field–induced polarizability anisotropy evolution from the MD. As detailed in the Supplementary Materials, our calculation is based on a dipole-induced dipole (DID) model for the FFMD (*31*). The most useful decomposition of the polarizability anisotropy in this case is to compare its value when intermolecular interactions are switched off (each molecule has its gas-phase polarizability) and when they are switched on through the interactions of each molecule with the dipoles of the surrounding molecules, the so-called collision-induced polarizability contribution. As shown in Fig. 4C, the total polarizability anisotropy of the whole simulation box shows a bipolar signal, which, to a large extent, resembles the TKE signal of water. In this calculation, the polarizability contains the contributions from the single molecules, as well as the collisions/interactions. Figure 4C also shows the result when the DID contribution is switched off, essentially the single-molecule picture. Here, the response is unipolar. This single versus total polarizability anisotropy analysis strongly suggests that the collision-induced polarizability is the main reason of the sign flip of the TKE response of liquid water pumped at ~1 THz.

### Energy dissipation

The main outcome of our MD simulations, shown in Fig. 4C, is that the observed dynamics in the TKE signal of water excited at ~1 THz originate from the collision/interaction-induced polarizability. The remaining intriguing question is the mechanism of this effect. A collision-induced change in the polarizability must eventually be traced back to the restricted rotations and translations of molecules, and how their polarizability is modified as they stumble into their neighbors while rotating/translating.

To shed light on the nature of the underlying motion, we refer again to our MD simulations and calculate the kinetic energy (KE) evolution of water molecules after their excitation with the THz pulse at ~1 THz. In the AIMD simulations, we partition the total KE of the system into three contributions from molecular rotational KE_rot_, translational KE_trans_, and intramolecular vibrational contributions. The latter component remains almost constant within the noise level, thereby is not shown. In the FFMD simulations, molecules are rigid and their KE has only rotational and translational contributions.

**Fig. 5:**
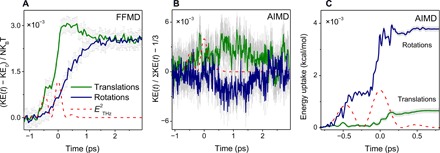
Molecular kinetic energy. (**A**) Temporal evolution of the average molecular translational (blue) and rotational (green) KE upon THz excitation, obtained from FFMD simulations. Here, the temperature rise of the system and the relaxation of the translational degrees of freedom are vividly observed. KE_0_ in the *y* axis is the averaged KE over 4 ps before applying the pulse. (**B**) Temporal evolution of the ratio of the molecular translational (green) and rational (blue) KE to the total instantaneous KE of the system from AIMD simulations. The deviation of the ratio from the equilibrium value of one-third is plotted so that a positive (negative) value indicates a relative increase (decrease) in the respective KE contribution in comparison to an equilibrium (equipartitioned) distribution; see also fig. S8 for the finite size impact of the MD simulation box on the MD results. (**C**) Total power deposited directly from the THz electric field into the molecular translations and rotations.

The FFMD simulations results, namely, KE_rot_(*t*) and KE_trans_(*t*), are depicted in Fig. 5. The figure shows that, while both KE_rot_ and KE_trans_ increase upon THz excitation, they remarkably differ in energy gain dynamics. KE_trans_ rises to its maximum value at *t* ≈ 0.2 ps, after which it relaxes to a temperature that is consistent with the expected temperature rise in the ~100-mK range. KE_rot_, on the other hand, appears to rise monotonically until the rotational-translational equipartitioning of energy is attained at higher temperature.

With the higher noise level in AIMD, the step-like rise in temperature cannot be seen. So here, we alternatively monitor the deviation from equipartitioning by plotting the ratio of the translational (rotational) KE to the instantaneous total KE. A deviation of this ratio from one-third gives a nonequilibrium distribution of KE. Here, we also observe the transient nonequilibrium partitioning of KE between the molecular translational and rotational dynamics, with KE_trans_ gaining more KE at the expense of KE_rot_. Note that the calculated ratio of the translational and rotational KEs to the instantaneous total KE from the FFMD approach (fig. S4) gives similar results as in the AIMD method, indicating the consistency of the MD results. The MD simulation results also show that the relaxation dynamics of the excess KE_trans_ match nicely with the relaxation tail of the TKE signal of water. In the FFMD, KE_trans_ relaxes exponentially with time constant τ ≈ 0.5 ps, and in AIMD, it relaxes to its equilibrium value also exponentially with a time constant of τ ≈ 0.75 ps.

Although the KE plot in Fig. 5A gives the impression that the translational motion originally gains THz energy and then is transferred to the rotations, further scrutiny shows that the energy transfer dynamics does not proceed this way. In the FFMD simulations, the electric field couples solely to the molecular rotations, as the water molecules are electrically neutral and there is no polarization or charge transfer between them. The H-bond stretching peak is absent from the IR spectrum based on nonpolarizable FFMD (*27*). Thereby, in the FFMD, only the rotational degrees of freedom acquire energy from the THz electric field, which is very rapidly transferred to the translations. The higher rise in translational KE might then be related to the rigidity and the stiffness of the binding between molecules, which causes the rapid energy transfer between rotations and the softer restricted translations.

The situation with AIMD is more subtle. Here, not only rotations but also translations are IR active. Previous AIMD simulations have shown that the H-bond stretching peak at ~200 cm^−1^ is IR active, while the H-bond bending peak at 50 cm^−1^ does not seem to be so (*27*). Here, to establish the energy flow pathway, we calculate the power absorbed from the THz electric field by the molecular translations and rotations. We accomplish this by directly calculating the coupling between the electric field vector and the molecular Born effective charges, in case of the translations and with the MD dipoles, for the rotations (*37*). As shown in Fig. 5C, integrating the accumulative power gives the total energy deposited in the translations/rotations (see the Supplementary Materials for details). There is some coupling between the pulse and molecular translations, which is about ~15% of the received energy from the THz pulse, but the majority of the energy, ~85%, is due to the field coupling to the rotational motions. We would like to stress that what is plotted in Fig. 5C is the deposited energy into either of the translational and rotational degrees of freedom to understand the coupling between the field and the system, which is very different from KE plot in Fig. 5A. The latter figure shows the energy exchange between rotations and translations superimposed on this energy uptake process.

The latter notion is also endorsed by our TKE experiment at ~3 THz. As shown in Fig. 1B, the pump pulse at ~3 THz has relatively large spectral overlap with the translational degrees of freedom of water. As a result, its unipolar TKE signal in Fig. 3B corroborates the fact that the direct excitation of the translational degrees of freedom does not lead to the bipolar TKE signal of water. The same behavior is observed in methanol, which is also a protic polar liquid with specific intermolecular interaction of H-bonding. As shown in fig. S5, excitation of the translational motion of methanol results on a unipolar TKE signal (*38*). Notably, by shifting the THz excitation frequency away from the translational degrees of freedom and toward its Debye processes with rotational origin (*39*), the bipolar TKE signal of methanol is resolved (*14*).

### Bipolarity of the TKE signal of liquid water

One remaining aspect that needs explanation is the nature of the collective motions that gives rise to the collision-induced polarizability and, consequently, the bipolar TKE response of water. One important hint is provided by the many-body expansion of water’s polarizability by Medders and Paesani (*40*). The authors showed that “both the total dipole and polarizability are almost entirely pairwise additive, with three-body terms contributing less than 4% and all higher-order terms being essentially negligible.” Thus, while we believe that the TKE is a collective phenomenon, like any emergent property, it can be understood by both the study of the behavior of the participating players and then also their relations and interactions among each other. In this regard, many-body expansions give at least a strong hint that low-order clusters might already provide the answer.

We also believe that the TKE signal of water and its peculiar bipolar shape have unique capacity to provide new microscopic insight on the collective dynamics associated with H-bonding network of water. A suggestive path would be the comparison between the measured TKE signal of water and the calculated Δ*n*_multimer_ = ΔΠ_multimer_ 〈*P*_2_( cos θ_multimer_)〉, based on the total polarizability anisotropy of water multimers and the alignment of their assigned dipole moment. As an example, we have provided a simple model based on a water dimer in the Supplementary Materials (see also figs. S9 and S10) by which the negative polarizability anisotropy of a collective entity such as a water dimer can be understood; however, rigorous theoretical studies are needed to provide more precise picture. Following this procedure, we will also likely be able to answer fundamental questions on the role of breaking and reforming of H-bonds in the transiently formed supramolecular structures in water.

The agreement between AIMD and FFMD results, shown in our study, suggests that the dynamics of water around 1 THz can be well reproduced with a rigid and nonpolarizable water potential. Moreover, the dynamic TKE of water can be simulated by the subsequent retracing of the force field trajectory using the DID model. However, the extension of this conclusion to the dynamics at higher frequencies, such as the O─O stretch vibration at ~5 THz, in which the intermolecular charge fluctuations are crucial (*27*, *41*), may not be straightforward.

In summary, upon resonant excitation of the low-frequency rotational motion of water molecules, a Raman response is observed, which is consistently ascribed to the restricted translational motion of water molecules. This response, which arises from the coupling of the intermolecular degrees of freedom of water, declares a pathway for the dissipation of external THz energy into the network of H-bonds. Our MD simulations corroborate this conclusion and show the increase of the KE of the molecular translational motion after the initial coupling of the THz electric field to the rotational motions. The ultrafast flow of energy in the H-bonding network of water may be explained by the strong anharmonicity of the interaction energy of the intermolecular degrees of freedom. Thereby, the TKE may be implemented to measure the efficiency of the rotational-translational energy transfer in aqueous solutions and open a new avenue to explore the impact of ions and biological macromolecules on the H-bonding structure of water (*42*).

## MATERIALS AND METHODS

### TKE experiment

In the experiment, the linearly polarized THz pump pulse is focused onto the sample cell. The induced transient birefringence is measured by a temporally delayed and collinearly propagating probe pulse whose incident linear polarization is set to an angle of 45° relative to the THz electric field. Because of the pump-induced birefringence, the probe field components polarized parallel (∥) and perpendicularl (⊥) to the pump field acquire a phase difference Δϕ when propagating through the sample, thereby resulting in elliptical polarization. Δϕ is detected with a combination of a quarter-wave plate and a Wollaston prism, which splits the incoming beam in two perpendicularly polarized beams with power *P*_∥_ and *P*_⊥_. In the limit ∣Δϕ∣ ≪ 1, the normalized difference *P*_∥_ − *P*_⊥_ fulfills

$$\frac{P_{∥}-P_{⊥}}{P_{∥}+P_{⊥}}\approx \Delta \phi$$ (2)

and is measured by two photodiodes as a function of the temporal delay between THz pump and probe pulse (*20*).

For the temperature-dependent TKE measurements, the static cell is attached to a Peltier element and the temperature of the liquid is calibrated in advance. The stability and accuracy of the liquid’s temperature is determined to be ±0.5 K.

### Temperature rise

To ensure that the accumulation of pump heat does not influence the results, we performed the TKE experiments also in a flow cell with the same SiN windows. We found no difference between static and flow cells in terms of both dynamics and amplitudes of the signals. Note that the simple calculations based on ∆*T* = *Q*/*mC*, with THz energy *Q* ≈ 6 μJ, the mass of the excited volume of water *m* ≈ 3 × 10^−5^ g, and heat capacity of water *C* = 4.18 J/g estimate a negligible temperature rise ∆*T* ≈ 50 mK.

The AIMD results also confirm that the temperature rise and the change in the H-bond density along the AIMD trajectory are negligible (see fig. S6). The H-bond survival probability (fig. S7) also shows no effect of the THz excitation on the lifetime of an H-bond. Therefore, the THz excitation in the experiment can be regarded as a small perturbation, which minimally distorts the H-bonded structure of water. After the pulse, we find a slight increase in the probability of H-bond being broken due to the translational diffusion of an initially H-bonded partner, and a slight decrease in the probability that an H-bond is broken because of rotational diffusion of an H-bond donor relative to the acceptor, with the effects cancelling each other so that the probability of survival of the H-bonds remains unaffected by the pulse.

*Note added in proof*: After this paper was accepted for publication, we became aware of a preprint, where the librational motion of liquid water was probed by THz-spectroscopy [arXiv:1809:04261].

## Supplementary Material

aay7074_SM.pdf

## SUPPLEMENTARY MATERIALS

Supplementary material for this article is available at http://advances.sciencemag.org/cgi/content/full/6/17/eaay7074/DC1
