# Supplementary material for: Energy transfer within the hydrogen bonding network of water following resonant terahertz excitation
Source: Sci Adv. 2020 Apr 24;6(17):eaay7074. doi: 10.1126/sciadv.aay7074 (PMC7182424; doi:10.1126/sciadv.aay7074)
Supplement: aay7074_SM.pdf [file aay7074_SM.pdf]

Science Advances
AAAS

advances.sciencemag.org/cgi/content/full/6/17/eaay7074/DC1

Supplementary Materials for

Energy transfer within the hydrogen bonding network of water following resonant terahertz excitation

Hossam Elgabarty, Tobias Kampfrath, Douwe Jan Bonthuis, Vasileios Balos, Naveen Kumar Kaliannan, Philip Loche, Roland R. Netz, Martin Wolf, Thomas D. Kühne*, Mohsen Sajadi*

*Corresponding author. Email: tdkuehne@mail.uni-paderborn.de (T.D.K.); sajadi@fhi-berlin.mpg.de (M.S.)

Published 24 April 2020, *Sci. Adv.* **6**, eaay7074 (2020)
DOI: 10.1126/sciadv.aay7074

This PDF file includes:

Molecular dynamics simulations–details
Bipolarity of the TKE signal of water
Figs. S1 to S10
References

Supplementary Materials

***Ab initio* Molecular dynamics simulations.** In this approach, the liquid is treated as a collection of nuclei and electrons to mimic the bulk conditions, under periodic boundary conditions. Molecular structure, interaction with the time-dependent electric field, and dielectric polarizability follow consistently from the electronic density of the quantum mechanical ground state as obtained within the Kohn-Sham formalism. Density functional theory-based *ab initio* MD simulations of a periodic cubic cell with 128 water molecules were performed at constant energy and ambient density (0.9966 g/cm3) using the second generation Car–Parrinello method (43). The energy and forces were computed using the mixed Gaussian-plane wave approach (44), where the Kohn–Sham orbitals were represented by an accurate triple-ζ basis set with two sets of polarization functions (TZV2P) (45), and plane-waves with cutoff of 400 Ry were used to represent the charge density. The BLYP exchange-correlation functional plus a damped interatomic potential to account for van der Waals interactions (Grimme-D3 (46)) were employed. Previous works have shown that this set-up provides a realistic description of many important structural, dynamical and spectroscopic characteristics of liquid water, including the partial pair correlation functions, self-diffusion and viscosity coefficients, HB lifetime, NMR shielding, as well as x-ray absorption and vibrational spectra (47). In addition to the static dielectric response of water to external electric fields of varying strengths (5). The system was equilibrated for 30ps before 225 de-correlated snapshots were extracted from a further 200ps segment of the equilibrated trajectory. Each snapshot was then used to start an individual trajectory under the effect of the same THz pulse profile and amplitude as used in our experiment. No thermostat was employed and all the theoretical results and figures reported herein are averaged over the 225 trajectories. We used a Berry-phase approach to ensure a proper description of the field under periodic boundary conditions (48). All computations were performed using the QUICKSTEP module of the CP2K suite of programs (49). For defining a hydrogen bond we used a simple geometric criterion (3.5 Å and 30 degrees (3).

Classical Molecular dynamics simulations. In the FFMD, the atomic pair interactions are parameterized using a combination of Lennard-Jones and Coulomb potentials. We use a rigid three-point water model (SPC/E) optimized to reproduce the density and structure of water (50). We simulate a box with 5360 SPC/E water molecules in the NVE ensemble using the GROMACS version 2018 (51). We use 3D periodic boundary conditions, a Lennard-Jones potential which is shifted by a constant such that it is zero at the cut-off at 0.9 nm. 3D Particle Mesh Ewald summation are applied for the electrostatics beyond 0.9 nm. At time $t_0 = 5$ ps we apply a THz pulse according to

$$E(t) = \frac{1}{2\pi\upsilon} E_0 \partial_t e^{-(t-t_0)^2/\sigma^2} \cos(2\pi\,\upsilon(t - t_0) + \varphi) \qquad \text{(S1)}$$

with $\upsilon = 0.5$/ps, $\sigma = 0.5$ ps = 0.5 ps, $\varphi = 0.628319$ and $E_0 = 1$ V/nm, which corresponds to a THz electric filed with a maximum amplitude of ~2 MV/cm (similar to the experimental one).

We calculate the total KE from the atomic velocities and the translational $\mathrm{KE_{trans}}$ from the velocity of the molecular centers of mass. The $\mathrm{KE_{rot}}$ is the difference between the two. During the simulation, the temperature increases on average less than 1 K. The curves shown in the paper are based on the average of 5000 trajectories of 50 ps, each containing a single pulse. We use a time step of 2 fs and a write-out frequency of 20 frames per ps.

We check for finite-size effects by simulating a water box of 128 molecules using the same procedure (see Fig. S8). As expected, the statistics are insufficient to draw any conclusions

when only a small number of pulses is averaged, but for 3200 pulses and above, the curves reproduce the results for the larger box size. This shows that finite-size effects are unimportant.

Kinetic energy decomposition. In the AIMD simulations, at each MD snapshot the decomposition was done for every molecule in the instantaneous molecular internal coordinates defined by the Eckart conditions (the Eckart frame), thus minimizing the vibrational-rotational (Coriolis) cross term (52). In the FFMD, the KE_{rot} is calculated as the difference between the total KE and the KE of the molecular centers of mass.

Calculation of polarizability anisotropy. In order to calculate the relaxation of polarizability anisotropy shown in Figure 6c in the main text, we have based our calculations on the dipole code developed by R. Khatib et al. (53) (code downloaded from github: ttps://github.com/RemiKhatib/dippol). We chose to use a single-point model for water's molecular dipole moment and polarizability, i.e. a point dipole and polarizability are placed at the center of mass of each water molecule, and oriented according to the molecular frame of reference. The polarizability of each molecule at each at each sampled MD time step is self-consistently calculated using the first-order dipole-induced dipole moment model. In this model, for each molecule i in the system one starts with it's gas phase dipole μ_i^0 and polarizability α_i^0, and then the induced polarizability (due to the electric field of the surrounding j molecules) is calculated from:

$$\alpha_i^{ind} = -\alpha_i^0 \ \sum_{\boldsymbol{j \neq i}} \boldsymbol{T}_{ij} \left(\alpha_j^0 + \alpha_i^{ind}\right) \qquad \text{(S2)}$$

where $\boldsymbol{T}_{ij}$ is the dipolar interaction tensor:

$$\boldsymbol{T}_{ij} = \frac{1}{\left|\boldsymbol{r}_{ij}\right|^3} - \frac{\boldsymbol{r}_{ij}.\boldsymbol{r}_{ji}}{\left|\boldsymbol{r}_{ij}\right|^5} \qquad \text{(S3)}$$

and a cutoff of 7.5 A° has been used to compute the induced polarizabilities. Obviously, the solution of equation (S2) requires knowledge of the induced polarizability of every other molecule in the system within the cutoff range, hence the equation is solved iteratively until self-consistency is achieved.

For α_0 we have used values obtained from accurate ab initio gas phase calculations at the coupled cluster level of theory (54). The polarizability anisotropy was calculated by first summing the polarizabilities of all the molecules in the laboratory frame at some time t, and then calculating $\Delta\alpha(t) = \alpha_{xx}(t) - (\alpha_{yy}(t) + \alpha_{zz}(t))/2$. The final result is obtained as an average over 5000 FFMD trajectories, each with 5360 water molecules.

As a validation of this model, we have compared the polarizability anisotropy values obtained using it, with those obtained from fully periodic ab initio Berry-phase calculations on snapshots extracted from the AIMD trajectories and averaged over all of them (55), and have found the results to be in agreement within the error bars of the AIMD.

Calculating the power exerted by the field on molecular translations and rotations.

In this section, we follow A. Pasquarello and R. Resta, Phys. Rev. B 68, 2003, 174302.

Translations

The power exerted an electric field on a charge moving with velocity $\boldsymbol{v}$ is simply given by the dot product of the electric force and the velocity $\boldsymbol{F} \cdot \boldsymbol{v}$. In the case of a point charge, the electric force is simply given by the electric field vector multiplied by the charge. For an isolated charge distribution with an overall zero charge, like a neutral molecule in vacuum, the electric field thus does not exert any work on the molecule when it translates, and the

lowest order interaction with the field would be with torque exerted on the dipole moment of the charge distribution, if the latter is not vanishing by symmetry.

For a molecule in condensed phase, even for a molecule that is formally neutral, things are different. Here, as a molecule translates, it interacts with its neighbors leading to distortions in the electronic distribution and polarization, which can lead to a non-vanishing coupling of the molecular translation with the applied field, as if the molecule is charged. Thus in this case, one can define a so-called dynamical molecular monopole in terms of the coupling between translational molecular modes and electric fields.

This dynamical monopole or Born effective charge, (Z) for some atom labelled i (Z_i) in a periodic system is defined as:

$$Z_{i,\alpha\beta} = \Omega \frac{\partial P_a}{\partial u_{i,\beta}} \quad \text{(S4)}$$

where Ω is the volume of the repeating periodical cell, and u_i is some Cartesian displacement of the atom along the β-axis. Z is thus given by a second-rank Cartesian tensor. An equivalent definition is:

$$F_{i,} = Z_{i,} E_{\beta} \quad \text{(S5)}$$

where F is the force induced on atomic nucleus i due to the electric field E, and Einstein summation notation is used. These dynamical charges are physical observables and measure the coupling between atomic displacements and electric fields.

In this work, we have used the dynamical monopoles of liquid water as reported by Pasquarello and Resta in the work cited in the previous paragraph. These were based on condensed phase calculations using DFT with the BLYP function, in close agreement with our AIMD setup. The total force exerted on a molecule is simply given by equation (S5) summed over all the atoms in the molecule. With the force at hand, we calculate the power exerted by the field on molecular translations, and integration of the latter quantity from time 0 to t gives the total work done on translations.

Rotations

The power exerted by the field on rotations of each molecules is given by the dot product of the electric torque with the molecular angular momentum. We calculate the molecular angular momentum in the Eckart frame, taking the molecular center of mass as the origin, and the electric torque (τ) is given by:

$$\tau_\alpha = \epsilon_{\alpha\gamma\delta} \sum_{atom=s} r_{s,\gamma} Z_{s,\beta\delta} E_\beta \quad \text{(S6)}$$

where $\epsilon_{\alpha\beta\gamma}\mu_\gamma$ is the Levi-Civita tensor, and $\boldsymbol{r_s}$ are the position vectors of the atomic nuclei in the molecule. Again, integration of the power yields the work done on rotations.

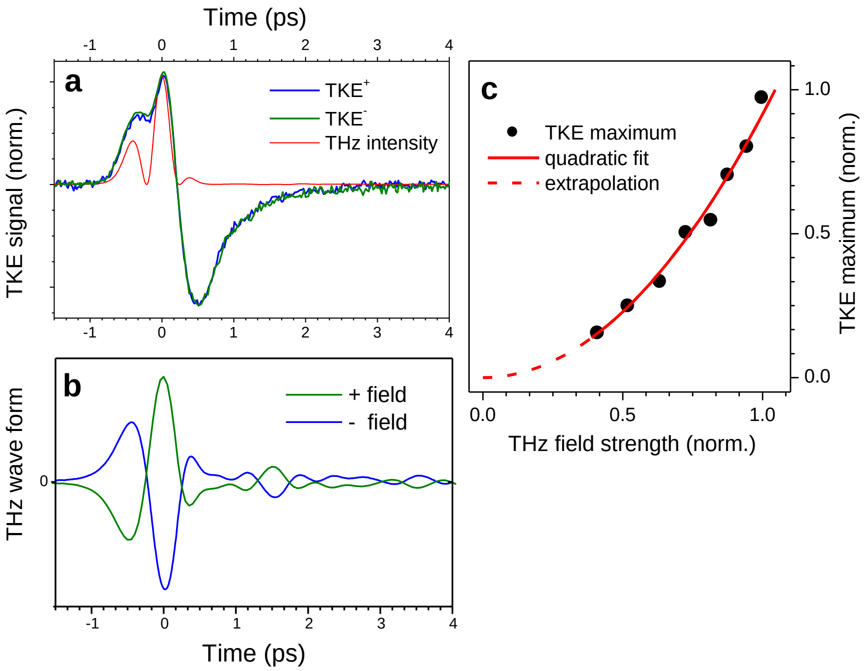

Figure S1 | THz Kerr effect of water. a, Induced optical birefringence of water (blue and green lines) by the THz electric fields with opposite polarity (see panel b) and the square of the driving THz field (red line). **b**, The wave forms of the THz electric fields with opposite polarities used in the experiment. **c**, Fluence dependence shows that the TKE of water scales quadratically with THz driving field.

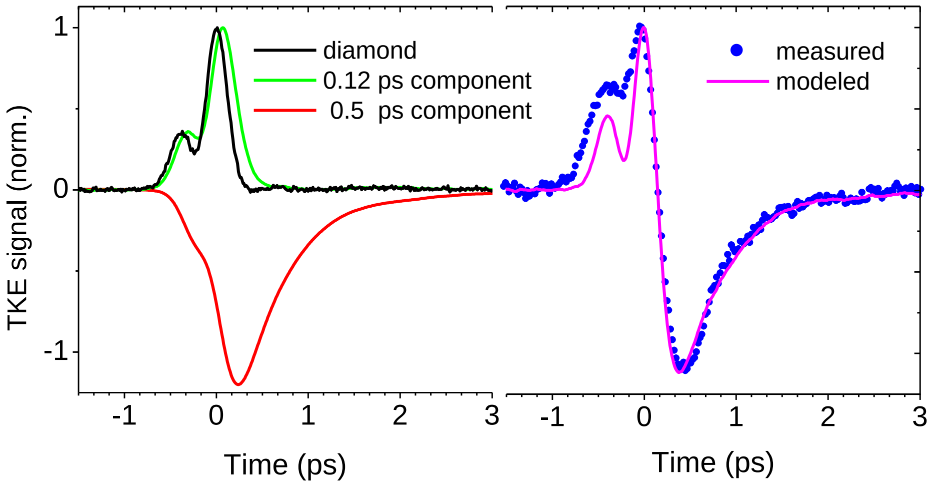

Figure S2 | Dynamic components of the bipolar TKE signal of water. left, Square of THz field (black line) is convoluted with two exponential functions with $\tau_1 \approx 0.12$ps (green line) and $\tau_2 \approx 0.5$ps (red line). right, The Sum of the latter two curves gives rise to the magenta line, which captures almost all features of the measured TKE signal of water (blue dots).

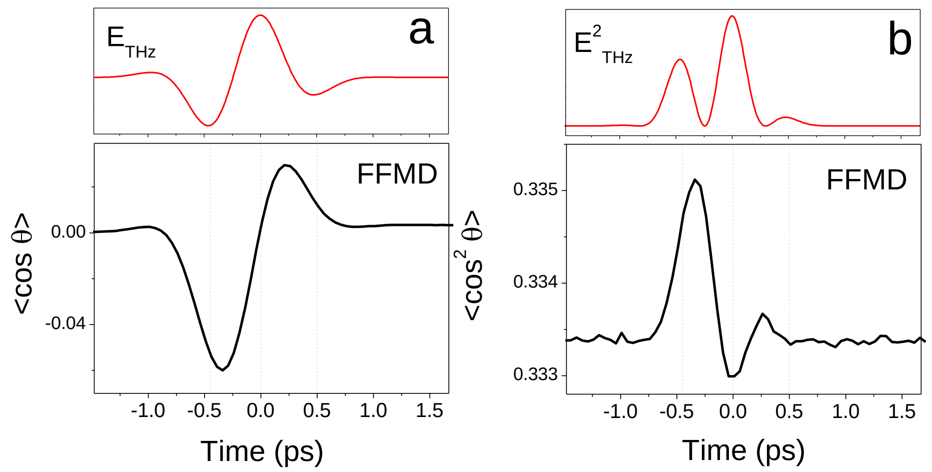

Figure S3 | Molecular orientation an alignment of liquid water. We repeat the calculation of the molecular orientation and alignment shown in Fig. 4a and Fig. 4b of the main text for a THz electric field with three times larger amplitude. The main findings are the same, however with much higher signal to noise ration.

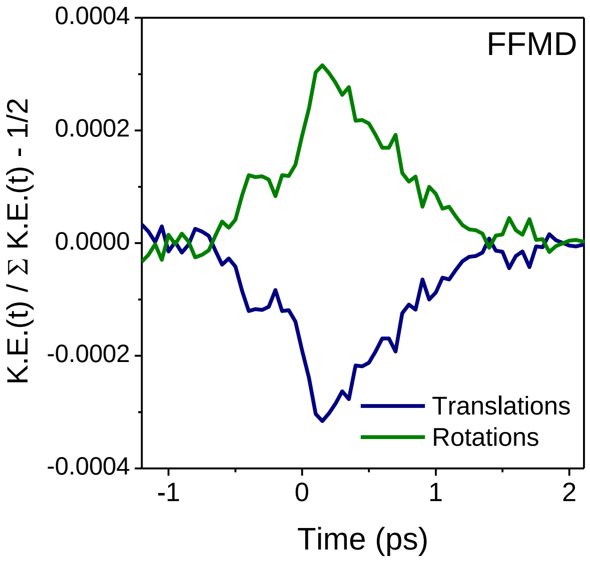

Figure S4 | Relative deviation from equipartition. The rotational and translational contributions to the kinetic energy given in Fig. 5a, FFMD results, are normalized to the instantaneous total kinetic energy, after which the equilibrium value 1/2 is subtracted.

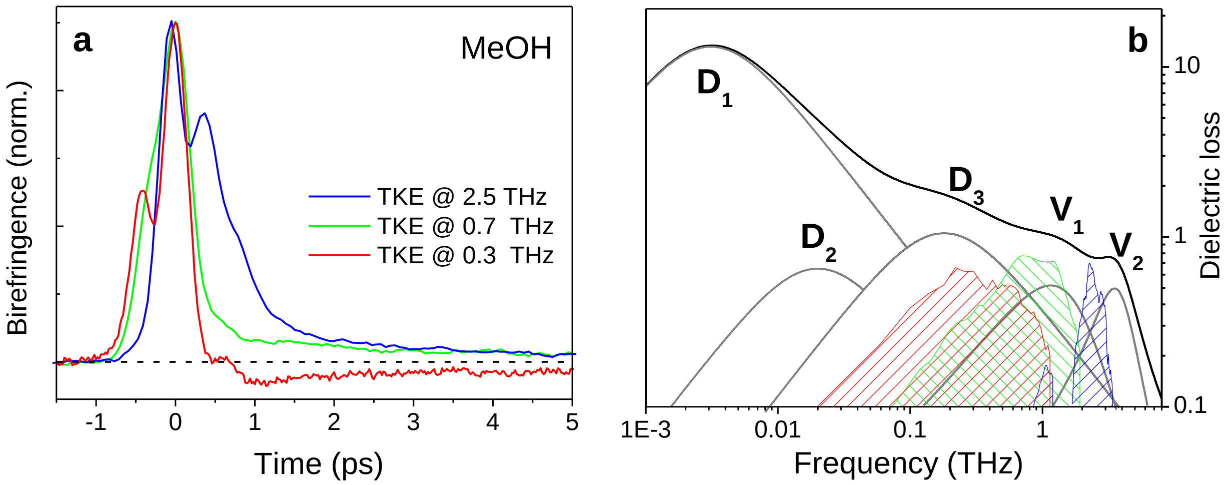

Fig. S5 | TKE of methanol. a) TKE signal of methanol is obtained after pumping the liquid with three different THz pulses whose central frequencies are ~0.3 THz, ~0.7 THz and ~3 THz. **b**) The Dielectric loss spectrum of methanol is commonly fit with three Debye processes and two vibrations. The dashed areas are the spectra of three THz pulses. Note that the red area which corresponds to the THz pulse with 0.3 THz central frequency has larger overall with Debye process of methanol and causes a bipolar TKE response.

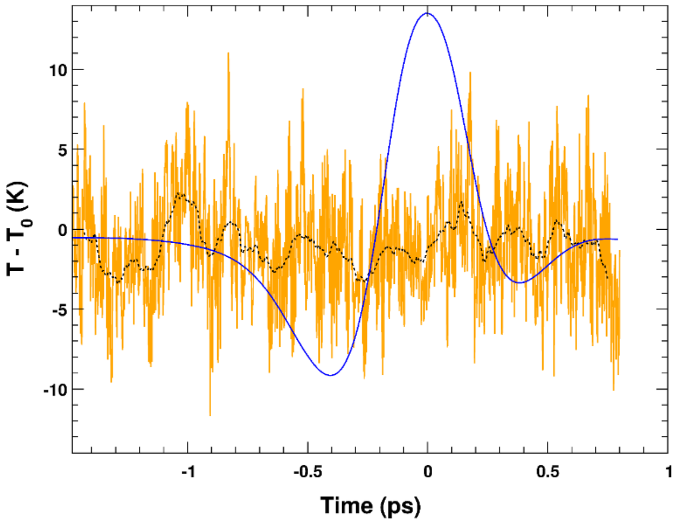

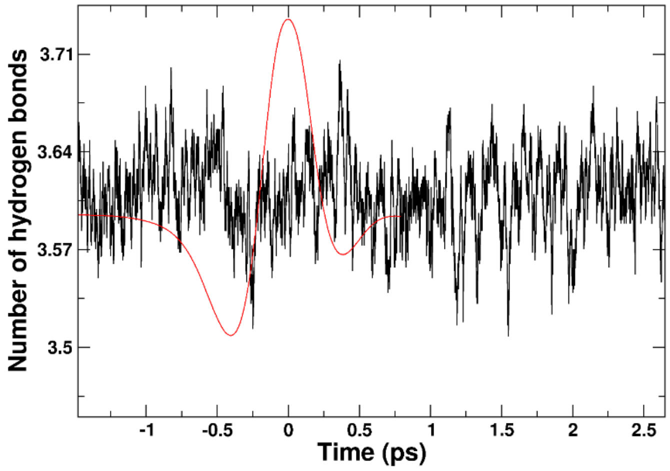

Figure S6 | Temperature rise and the number of hydrogen bond per water molecule. up, Ab initio molecular dynamics simulations resolves almost no change in the temperature of water after the THz excitation within its noise level. Here T_0 is the ambient temperature. The black dots represent a 100 fs running average. down, The number of H-bonds remain almost constant even after THz excitation.

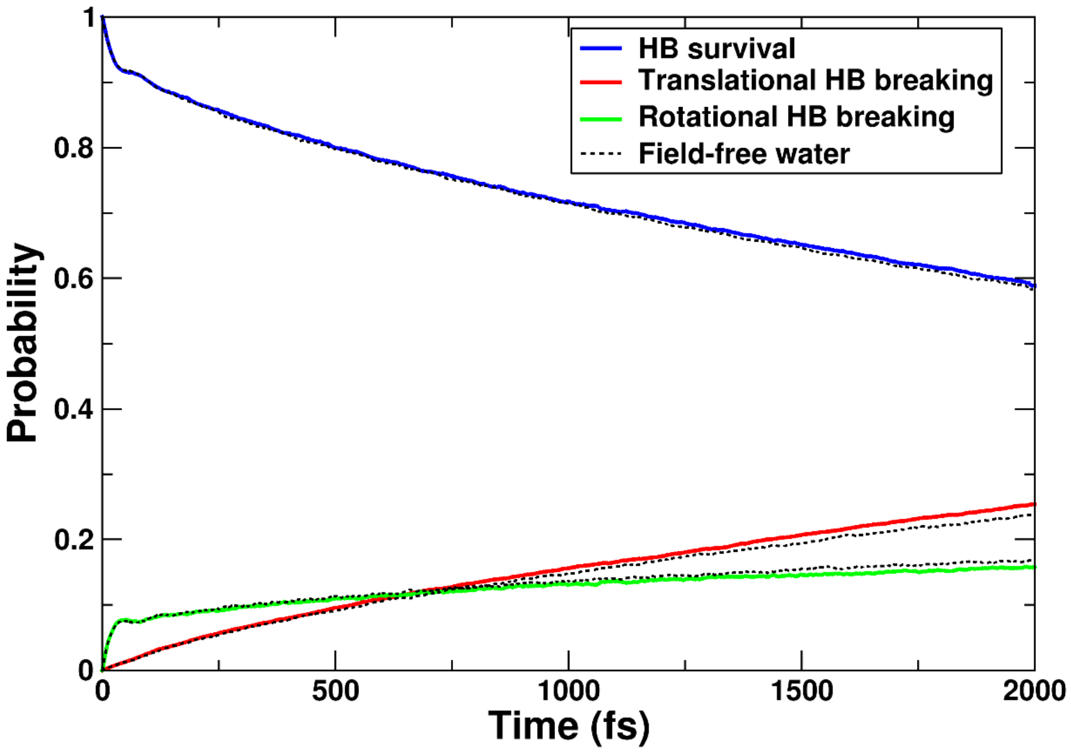

Figure S7 | Survival probability of H-bonds. Blue: The probability for a hydrogen bond that was present at an initial time t=0 (which we choose to coincide with the first peak in the pulse) to survive at later times. For those hydrogen bonds that are broken we have also calculated the probability that the hydrogen bond is broken due to translational (red, see illustration at bottom left) or rotational (green, see illustration at bottom right) motion of the hydrogen bond acceptor relative to the hydrogen bond donor. In all cases the dotted lines show the same quantities for an equilibrium microcanonical field-free trajectory, which shows that, while the H-bond lifetime remains unaffected by the pulse, there is an increase in the contribution of the translational motion to the H-bond breaking events which is compensated for by a decrease in the rotational contribution. In this plot we define a hydrogen bond to have an O–O distance < 3.5 Å, and an angle of < 30°. A hydrogen bond is considered broken once these criteria are violated for a continuous time segment longer than 20 fs.

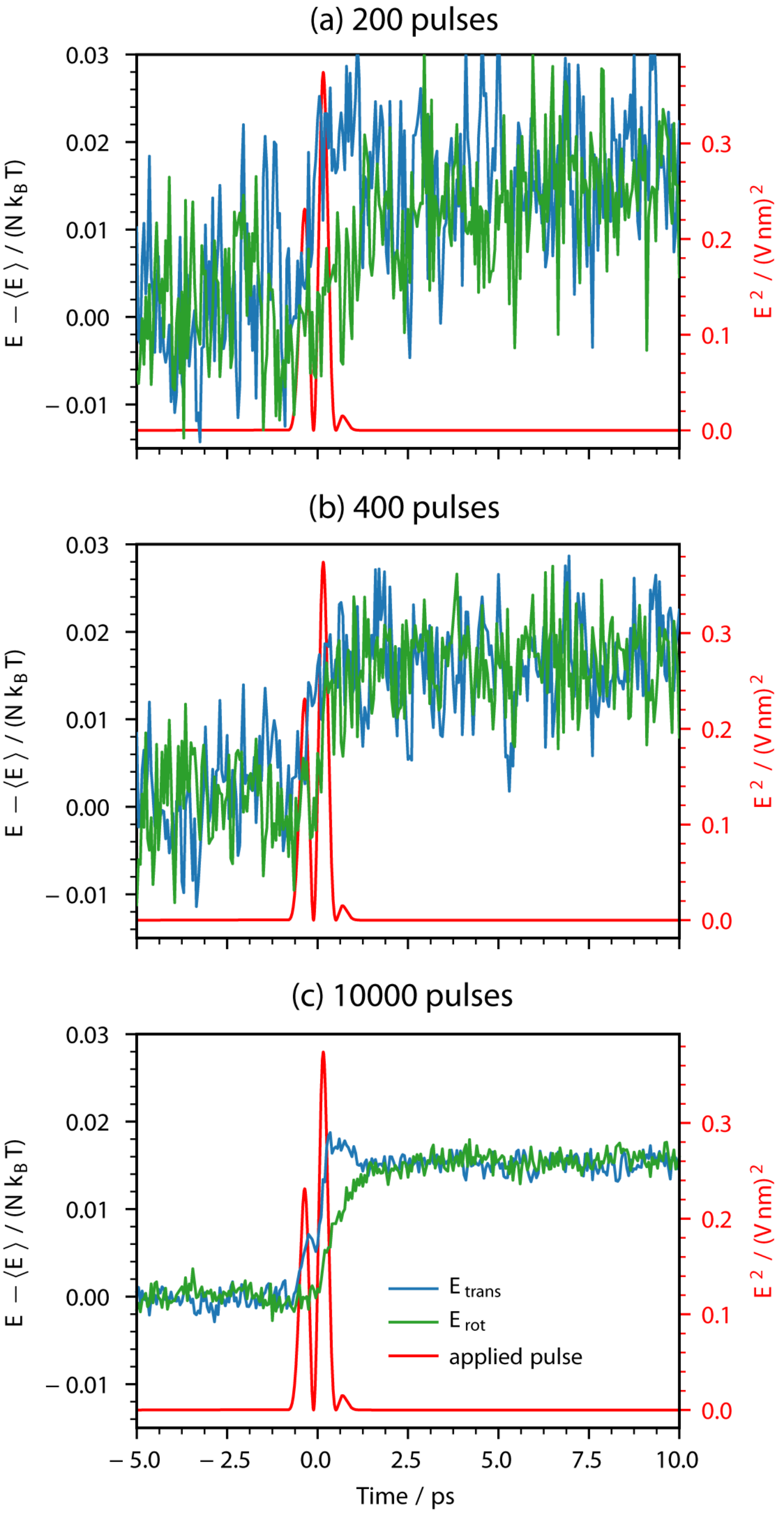

Figure S8 | Impact of the finite size of the MD simulation box. Rotational and translational kinetic energy calculated in FFMD simulations using 128 water molecules, averaged over different numbers of pulses. The result for 10000 pulses agrees with the results obtained using 5360 water molecules (Fig. 5 of the main text), showing that finite-size effects are negligible. Here, the applied THz field has the amplitude of $E_0 = 2.5\ \mathrm{V/nm}$ (see Eq. S1) to gain higher signal to noise ratio.

Bipolarity of the TKE signal. As mentioned in the main text, the prerequisite for observing a bipolar TKE signal of an isolated molecule is its negative polarizability anisotropy, i.e. the polarizability along the dipole axis is smaller than the polarizability average in the plane perpendicular to this axis. As this criterion isn't fulfilled for single water molecules, we go beyond the single molecule intrinsic polarizability and envisage low-order clusters, which is in the simplest case a dimer, to include the impact of the neighboring molecules to the total polarizability of water.

As highlighted in Fig. S10, the partial O-H group of molecule A can be regarded as the permanent dipole of the dimer. By applying a THz torque to the OH dipole, the energy of this coupling is transferred to its neighboring molecule B and increases its translational kinetic energy. Consequently, the latter excess kinetic energy leads to the stronger collision of molecule B with its adjacent neighbors, resulting in the deformation of its electron cloud. The latter electron cloud deformation is indeed the additional collision-induced polarizability contribution to the total polarizability of the dimer. Indeed, the translational motion of molecule B can be decomposed to its components along and perpendicular to the OH dipole. The latter of which corresponds to the motion of molecule B in the normal mode coordinate of H-bond bending mode, and the former matches the coordinate of the stretch vibration.

We suggest that molecule B gains more translational kinetic energy for its motion in the H-bonding mode coordinate because of the spectral vicinity of this mode and the excited region. Moreover, the H-bond bending has a pronounced amplitude in the Raman response of water at 1-2 THz frequency range and its expected dynamic Kerr response would decay with $\tau = (\frac{c\gamma_{\mathrm{B}}}{2})^{-1} \approx 0.57\mathrm{ps}$ (c is the speed of light), very close to the relaxation time of the observed bipolar TKE response of water. This intermediate relaxation time has also been resolved in previous OKE experiments and similarly assigned to the relaxation of the H-bond bending of water (56,57). Moreover, our temperature-dependent TKE measurement does not contradict this assignment. As shown in Fig. S9a and Fig. S9b, the magnitude of the T-dependent TKE decay time has the closest affinity to the T-dependence of the relaxation of the H-bond bending mode (58), relative to the other relaxation processes of water.

Accordingly, the collision-induced polarizability of the dimer perpendicular to the O-H dipole becomes larger than its parallel component. As a result, the polarizability anisotropy of the dimer can be conceived as negative $\Delta\Pi^{\mathrm{I}} = \Pi^{\mathrm{I}}_{\parallel} - \Pi^{\mathrm{I}}_{\perp} < 0$. In this simplified picture, the dimer is the rotating entity whose response $\Delta n_{\mathrm{rot-dimer}} \propto \Delta\Pi^{\mathrm{I}}\langle P_2(\cos\theta_{\mathrm{dimer}})\rangle$ is resolved in the TKE experiment. Note that the dimer picture tends to be a toy model to provide a simple understanding of the bipolar TKE signal of water. Obviously more rigorous studies are required to shed light on the collective intermolecular dynamics of water (59).

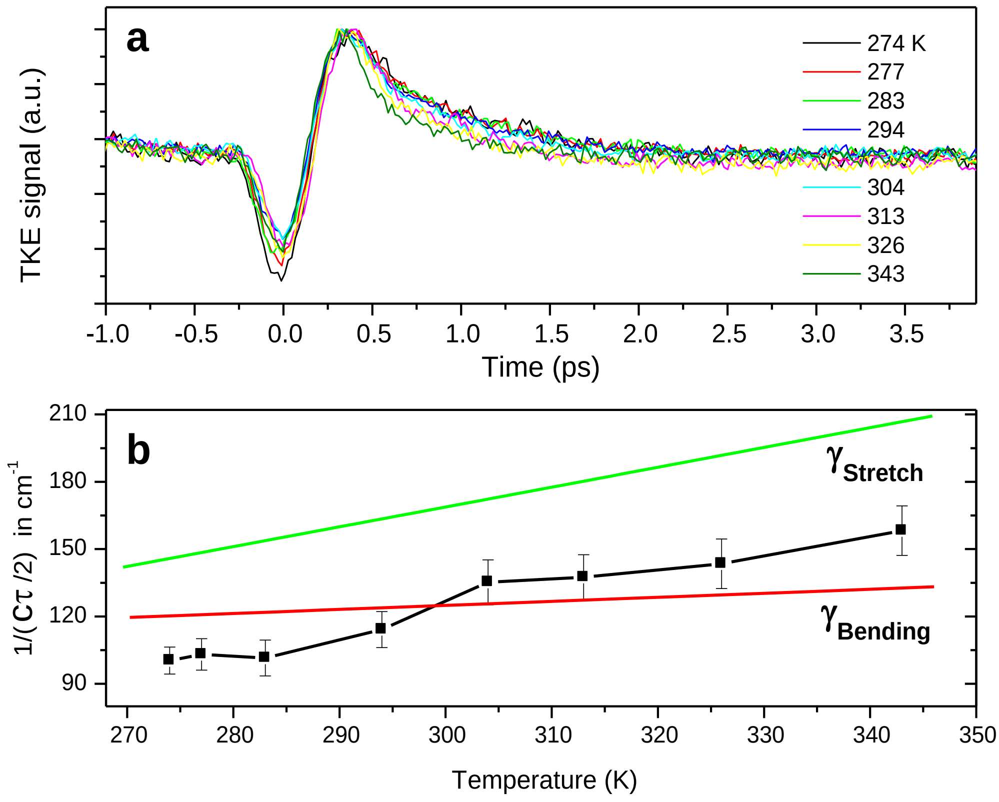

Figure S9 | TKE of water-temperature dependence. a, By increasing the temperature of water, the relaxation tail of the TKE signal becomes faster. All signals were recorded in one set of measurement. **b**, The red and green curves show the change of the bandwidths of the H-bond bending and O-O stretch vibration modes of water drawn from the Fig. 5 of Ref. 58 and the black square-line shows variation of the relaxation time of the TKE signal of water as function of time. Here the y-axis is $1/(c\tau/2)$, where τ is the relaxation time of the TKE signal and c is the speed of light. The error bars in the panel b shows only the root mean square deviation of the relaxation tail to a single exponential fit, other possible errors in the measurement are not considered here. Note that the relaxation of the fast Debye process of water (D_2 in **Fig. 1b**), which has a large spectral overlap with the THz pump spectrum, is almost temperature independent (25).

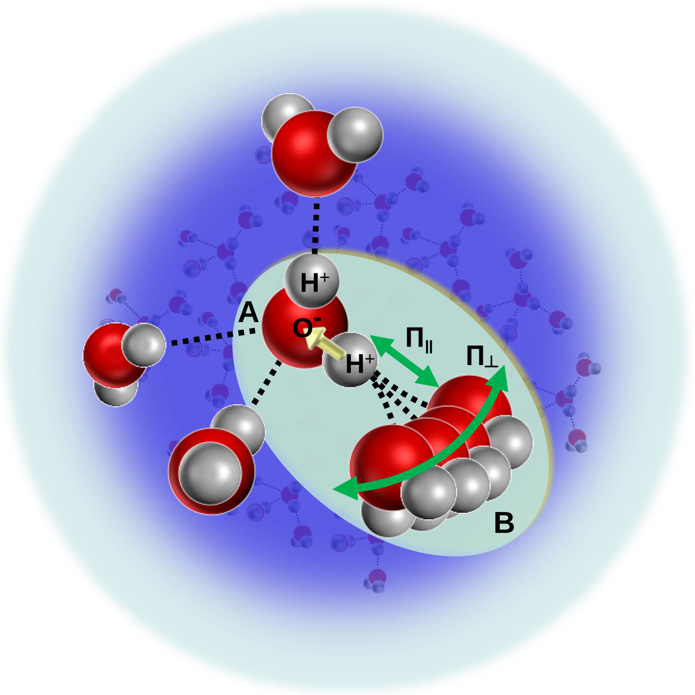

Figure S10 | Impact of collision induce polarizability in TKE signal. To explain the sign flip of the TKE signal of water, we suggest a simple dimer model. In the highlighted dimer structure, the permanent dipole is given by the O-H group of molecule A. Upon the coupling of the THz electric-field to the latter dipole, the energy of this interaction is transferred to the molecule B and increases its kinetic energy (facilitated by the H-bonding between the two neighbors). As discussed in the text above, for the excitation frequency ~1 THz, the lateral motion of molecule B perpendicular to the OH dipole (along the normal mode coordinate of H-bond bending mode), is more likely to gain larger kinetic energy rise, relative to the translational motion along the OH dipole. As a result, the electron cloud of molecule B with excess kinetic energy is deformed, because of its collision with adjacent neighbors. The latter deformation of electron cloud would then be the origin of the larger polarizability of the dimer perpendicular to the OH dipole relative to the polarizability component along the OH dipole axis, such that $\Delta\Pi^{\mathrm{I}} = \Pi_{\parallel}^{\mathrm{I}} - \Pi_{\perp}^{\mathrm{I}} < 0$. Accordingly, the TKE response of this dimer $\Delta n_{\mathrm{rot-dimer}} \propto \Delta\Pi^{\mathrm{I}}\langle P_2(\cos\theta_{\mathrm{dimer}})\rangle$ is negative.

REFERENCES AND NOTES

1. J. B. Hasted, *Water: A Comprehensive Treatise*, F. Franks, Ed. (Plenum Press, 1972).

2. S. V. Lishchuk, N. P. Malomuzh, P. V. Makhlaichuk, Contribution of H-bond vibrations to heat capacity of water. *Phys. Lett. A* **375**, 2656–2660 (2011).

3. A. Luzar, D. Chandler, Hydrogen-bond kinetics in liquid water. *Nature* **379**, 55–57 (1996).

4. T. D. Kühne, M. Krack, M. Parrinello, Static and dynamical properties of liquid water from first principles by a novel Car−Parrinello-like approach. *J. Chem. Theory Comput.* **5**, 235–241 (2009).

5. H. Elgabarty, N. K. Kaliannan, T. D. Kühne, Enhancement of the local asymmetry in the hydrogen bond network of liquid water by an ultrafast electric field pulse, *Sci. Rep.* **9**, 10002 (2019).

6. R. M. Whitnell, K. R. Wilson, J. T. Hynes, Vibrational relaxation of a dipolar molecule in water. *J. Chem. Phys.* **96**, 5354–5369 (1992).

7. K. Trachenko, V. V. Brazhkin, Collective modes and thermodynamics of the liquid state. *Rep. Prog. Phys.* **79**, 016502 (2016).

8. J. R. Errington, P. G. Debenedetti, Relationship between structural order and the anomalies of liquid water. *Nature* **409**, 318–321 (2001).

9. S. Woutersen, H. J. Bakker, Resonant intermolecular transfer of vibrational energy in liquid water. *Nature* **402**, 507–509 (1999).

10. M. L. Cowan, B. D. Bruner, N. Huse, J. R. Dwyer, B. Chugh, E. T. J. Nibbering, T. Elsaesser, R. J. D. Miller, Ultrafast memory loss and energy redistribution in the hydrogen bond network of liquid H_2O. *Nature* **434**, 199–202 (2005).

11. S. Ashihara, N. Huse, A. Espagne, E. T. J. Nibbering, T. Elsaesser, Ultrafast structural dynamics of water induced by dissipation of vibrational energy. *J. Phys. Chem. A* **111**, 743–746 (2007).

12. I. A. Finneran, R. Welsch, M. A. Allodi, T. F. Miller III, G. A. Blake, Coherent two-dimensional terahertz-terahertz-Raman spectroscopy. *Proc. Natl. Acad. Sci. U.S.A.* **113**, 6857–6861 (2016).

13. M. Sajadi, M. Wolf, T. Kampfrath, Transient birefringence of liquids induced by terahertz electric-field torque on permanent molecular dipoles. *Nat. Commun.* **8**,14963 (2017).

14. P. Zalden, L. Song, X. Wu, H. Huang, F. Ahr, O. D. Mücke, J. Reichert, M. Thorwart, P. K. Mishra, R. Welsch, R. Santra, F. X. Kärtner, C. Bressler, Molecular polarizability anisotropy of liquid water revealed by terahertz-induced transient orientation. *Nat. Commun.* **9**, 2142 (2018).

15. P. K. Mishra, V. Bettaque, O. Vendrell, R. Santra, R. Welsch, Prospects of using high-intensity THz pulses to induce ultrafast temperature-jumps in liquid water, *J. Phys. Chem. A* **122**, 5211–5222 (2018).

16. N. J. English, C. J. Waldron,Perspectives on external electric fields in molecular simulation: progress, prospects and challenges. *Phys. Chem. Chem. Phys.* **17**, 12407–12440 (2015).

17. J. Savolainen, S. Ahmed, P. Hamm, Two-dimensional Raman-terahertz spectroscopy of water. *Proc. Natl. Acad. Sci. U.S.A.* **110**, 20402–20407 (2013).

18. M. C. Hoffmann, N. C. Brandt, H. Y. Hwang, K.-L. Yeh, K. A. Nelson, Terahertz Kerr effect. *Appl. Phys. Lett.* **95**, 231105 (2009).

19. S. Sarbak, G. Sharma, C. S. Joseph, W. E. Kucia, K. Dobek, R. H. Giles, A. Dobek, Direct observation of the THz Kerr effect (TKE) in deionized, distilled and buffered (PBS) water. *Phys. Chem. Chem. Phys.* **19**, 26749–26757 (2017).

20. M. Sajadi, M. Wolf, T. Kampfrath, Terahertz-field-induced optical birefringence in common window and substrate materials. *Opt. Express* **23**, 28985–28992 (2015).

21. H. Hirori, A. Doi, F. Blanchard, K. Tanaka, Single-cycle terahertz pulses with amplitudes exceeding 1 MV/cm generated by optical rectification in $LiNbO_3$. *Appl. Phys. Lett.* **98**, 091106-3 (2011).

22. C. P. Hauri, C. Ruchert, C. Vicario, F. Ardana, Strong-field single-cycle THz pulses generated in an organic crystal. *App. Phys. Lett.* **99**, 1611163 (2011).

23. A. Sell, A. Leitenstorfer, R. Huber, Phase-locked generation and field-resolved detection of widely tunable terahertz pulses with amplitudes exceeding 100 MV/cm. *Opt. Lett.* **33**, 2767–2769 (2008).

24. T. Kampfrath, M. Wolf, M. Sajadi, Anharmonic coupling between intermolecular motions of water revealed by terahertz Kerr effect. arXiv:1707.07622v1 [cond-mat.soft] (24 July 2017).

25. H. Yada, M. Nagai, K. Tanaka, Origin of the fast relaxation component of water and heavy water revealed by terahertz time-domain attenuated total reflection spectroscopy. *Chem. Phys. Lett.* **464**, 166–170 (2008).

26. T. Fukasawa, T. Sato, J. Watanabe, Y. Hama, W. Kunz, R. Buchner, Relation between dielectric and low-frequency Raman spectra of hydrogen-bond liquids. *Phys. Rev. Lett.* **95**, 197802-4 (2005).

27. M. Sharma, R. Resta, R. Car. Intermolecular dynamical charge fluctuations in water: A signature of the H-Bond Network. *Phys. Rev. Lett.* **95**, 187401 (2005).

28. P. L. Silvestrelli, M. Bernasconi, M. Parrinello, Ab initio infrared spectrum of liquid water. *Chem. Phys. Lett.* **277**, 478–482 (1997).

29. R. Buchner, J. Barthel, J. Stauber, The dielectric relaxation of water between 0°C and 35°C. *Chem. Phys. Lett.* **306**, 57–63 (1999).

30. R. W. Boyd, *Nonlinear Optics* (Academic Press, ed. 3, 2007)

31. M. S. Skaf, S. M. Vechi, Polarizability anisotropy relaxation in pure and aqueous dimethylsulfoxide, *J. Chem. Phys.* **119**, 2181–2187 (2003).

32. S. Fleischer, Y. Zhou, R. W. Field, K. A. Nelson, Molecular orientation and alignment by intense single-cycle THz pulses. *Phys. Rev. Lett.* **107**, 163603 (2011).

33. T. Kampfrath, M. Wolf, M. Sajadi, The sign of the polarizability anisotropy of polar molecules is obtained from the terahertz Kerr effect. *Chem. Phys. Lett.* **692**, 319–323 (2018).

34. B. Bosma, L. E. Fried, S. Mukamel, Simulation of the intermolecular vibrational spectra of liquid water and water clusters. *J. Chem. Phys.* **98**, 4413–4421 (1993).

35. X. Ge, D. Lu, Molecular polarizability of water from local dielectric response theory. *Phys. Rev. B* **96**, 075114 (2017).

36. R. Buchner, What can be learnt from dielectric relaxation spectroscopy about ion solvation and association? *Pure Appl. Chem.* **80**, 1239–1252 (2008).

37. A. Pasquarello, R. Resta, Dynamical monopoles and dipoles in a condensed molecular system: The case of liquid water. *Phys. Rev. B* **68**, 174302 (2003).

38. T. Kampfrath, R. K. Campen, M. Wolf, M. Sajadi, The nature of the dielectric response of methanol revealed by the terahertz Kerr effect, *J. Phys. Chem. Lett.* **9**, 1279–1283 (2018).

39. J. Barthel, K. Bachhuber, R. Buchner, H. Hetzenauer, Dielectric spectra of some common solvents in the microwave region. Water and lower alcohols. *Chem. Phys. Lett.* **165**, 369–373 (1990).

40. G. R. Medders, F. Paesani, A critical assessment of Two-Body and Three-Body interactions in water. *J. Chem. Theory Comput.* **9**, 4844–4852 (2013).

41. E. Harder, J. D. Eaves, A. Tokmakoff, B. J. Berne, Polarizable molecules in the vibrational spectroscopy of water. *Proc. Natl. Acad. Sci. U.S.A.* **102**, 11611–11616 (2005).

42. R. C. Remsing, T. T. Duignan, M. D. Baer, G. K. Schenter, C. J. Mundy, J. D. Weeks, Water lone pair delocalization in classical and quantum descriptions of the hydration of model ions. *J. Phys. Chem. B* **122**, 3519–3527 (2018).

43. T. D. Kühne, Second generation Car–Parrinello molecular dynamics. *WIREs Comput. Mol. Sci.* **4**, 391–406 (2014).

44. G. Lippert, J. Hutter, M. Parrinello, A hybrid Gaussian and plane wave density functional scheme. *Mol. Phys.* **92**, 477–488 (1997).

45. J. VandeVondele, J. Hutter, Gaussian basis sets for accurate calculations on molecular systems in gas and condensed phases. *J. Chem. Phys.* **127**, 114105 (2007).

46. S. Grimme, J. Antony, S. Ehrlich, H. Krieg, A consistent and accurate *ab initio* parametrization of density functional dispersion correction (DFT-D) for the 94 elements H-Pu. *J. Chem. Phys.* **132**, 154104 (2010).

47. H. Elgabarty, R. Z. Khaliullin, T. D. Kühne, Covalency of hydrogen bonds in liquid water can be probed by proton nuclear magnetic resonance experiments. *Nat. Commun.* **6**, 8318 (2015).

48. I. Souza, J. Íñiguez, D. Vanderbilt, First-principles approach to insulators in finite electric fields. *Phys. Rev. Lett.* **89**, 117602 (2002).

49. J. VandeVondele, M. Krack, F. Mohamed, M. Parrinello, T. Chassaing, J. Hutter, Quickstep: Fast and accurate density functional calculations using a mixed Gaussian and plane waves approach. *Comput. Phys. Commun.* **167**, 103–128 (2005).

50. H. J. C. Berendsen, J. R. Grigera, T. P. Straatsma, The missing term in effective pair potentials. *J. Phys. Chem.* **91**, 6269–6271 (1987).

51. M. J. Abraham, T. Murtola, R. Schulz, S. Páll, J. C. Smith, B. Hess, E. Lindahl, GROMACS: High performance molecular simulations through multi-level parallelism from laptops to supercomputers. *SoftwareX* **1–2**, 19–25 (2015).

52. J. D. Louck, H. W. Galbraith, Eckart vectors, Eckart frames, and polyatomic molecules. *Rev. Mod. Phys.* **48**, 69–106 (1976).

53. R. Khatib, T. Hasegawa, M. Sulpizi, E. H. G. Backus, M. Bonn, Y. Nagata, Molecular dynamics simulations of SFG librational modes spectra of water at the water–air interface, *J. Phys. Chem. C* **120**, 18665–18673 (2016).

54. R. Bukowski, K. Szalewicz, G. C. Groenenboom, A. van der Avoird, Polarizable interaction potential for water from coupled cluster calculations. I. Analysis of dimer potential energy surface, *J. Chem. Phys.* **128**, 094313 (2008).

55. S. Luber, M. Iannuzzi, J. Hutter, Raman spectra from *ab initio* molecular dynamics and its application to liquid *S*-methyloxirane. *J. Chem. Phys.* **141**, 094503 (2014).

56. K. Winkler, J. Lindner, H. Bursing, P. Vöhringer, Ultrafast Raman-induced Kerr-effect of water: Single molecule versus collective motions. *J. Chem. Phys.* **113**, 4674–4682 (2000).

57. K. Winkler, J. Lindner, P. Vöhringer, Low-frequency depolarized Raman-spectral density of liquid water from femtosecond optical Kerr-effect measurements: Lineshape analysis of restricted translational modes. *Phys. Chem. Chem. Phys.* **4**, 2144–2155 (2002).

58. K. Mizoguchi, Y. Hori, Y. Tominaga, Study on dynamical structure in water and heavy water by low-frequency Raman spectroscopy. *J. Chem. Phys.* **97**, 1961–1968 (1992).

59. M. Heyden, J. Sun, S. Funkner, G. Mathias, H. Forbert, M. Havenith, D. Marx, Dissecting the THz spectrum of liquid water from first principles via correlations in time and space. *Proc. Natl. Acad. Sci. U.S.A.* **107**, 12068–12073 (2010).
